# Pulmonary resection for NSCLC in octogenarians: a single center experience

**DOI:** 10.1186/1471-2318-11-S1-A11

**Published:** 2011-08-24

**Authors:** S Di Russo, A Mani, L Guetti, P Camplese, G Cipollone, T Iarussi, F Mucilli

**Affiliations:** 1 Department of Surgical Sciences – Unit of Surgical Clinic, University of Chieti “G. D’Annunzio”, Chieti, Italy

## Background

Lung cancer is the second leading cancer death among octogenarians [^1^] and in particular NSCLC accounts for more than 90% of all lung cancers. Since life expectancy is improving, lung cancers are observed among octogenarians people more often. Surgical resection remains the treatment of choice for early stage lung cancer^2^. The authors report the outcomes of pulmonary resection for NSCLC in octogenarians.

## Materials and methods

We reviewed our center experience in elderly patients between 1999 and 2009 and reported perioperative mortality and morbidity. Thirty patients older than 80 years underwent surgical resection for NSCLC. Among these 19 lobectomies, 1 sleeve-lobectomy, 2 bilobectomies, 6 segmentectomies and 2 wedge resections were performed.

## Results

Post-operative non fatal complication occurred in 6 patients (20%): atrial fibrillation in 3 patients (10%), blood loss in 2 patients (6.6%), renal failure in 2 patients (6.6%), transitory cardiac arrhythmia in 1 patient (3%) and respiratory failure in 1 patient (3%). There was 1 post-operative death (3%).

## Conclusions

The advances in preoperative and postoperative care and surgical technique allow a larger number of curative resections in octogenarians patients [^2^]. Therefore age is not an absolute contraindication for NSCLC surgical treatment but pre-operative study is mandatory. It highlights comorbidity that can badly influence the post-operatory outcome and the survival.
